# Monitoring of Cardiorespiratory Signals Using Thermal Imaging: A Pilot Study on Healthy Human Subjects

**DOI:** 10.3390/s18051541

**Published:** 2018-05-13

**Authors:** Carina Barbosa Pereira, Michael Czaplik, Vladimir Blazek, Steffen Leonhardt, Daniel Teichmann

**Affiliations:** 1 Chair for Medical Information Technology, Helmholtz Institute for Biomedical Engineering, RWTH Aachen University, Pauwelsstr. 20, D-52074 Aachen, Germany; blazek@hia.rwth-aachen.de (V.B.); leonhardt@hia.rwth-aachen.de (S.L.); teichmann@hia.rwth-aachen.de (D.T.); 2 Department of Anesthesiology, University Hospital RWTH Aachen, Pauwelsstr. 30, D-52074 Aachen, Germany; mczaplik@ukaachen.de; 3 Czech Institute of Informatics, Robotics and Cybernetics (CIIRC), CTU Prague, Zikova street 1903/4, 166 36 Prague, Czech Republic

**Keywords:** thermal imaging, infrared thermography, infrared imaging, contactless measurement, heart rate, respiratory rate

## Abstract

Heart rate (HR) and respiratory rate (RR) are important parameters for patient assessment. However, current measurement techniques require attachment of sensors to the patient’s body, often leading to discomfort, stress and even pain. A new algorithm is presented for monitoring both HR and RR using thermal imaging. The cyclical ejection of blood flow from the heart to the head (through carotid arteries and thoracic aorta) leads to periodic movements of the head; these vertical movements are used to assess HR. Respiratory rate is estimated by using temperature fluctuations under the nose during the respiratory cycle. To test the viability and feasibility of this approach, a pilot study was conducted with 20 healthy subjects (aged 18–36 and 1 aged 50 years). The study consisted of two phases: phase A (frontal view acquisitions) and phase B (side view acquisitions). To validate the results, photoplethysmography and thoracic effort (piezoplethysmography) were simultaneously recorded. High agreement between infrared thermography and ground truth/gold standard was achieved. For HR, the root-mean-square errors (RMSE) for phases A and B were 3.53 ± 1.53 and 3.43 ± 1.61 beats per minute, respectively. For RR, the RMSE between thermal imaging and piezoplethysmography stayed around 0.71 ± 0.30 breaths per minute (phase A). This study demonstrates that infrared thermography may be a promising, clinically relevant alternative for the assessment of HR and RR.

## 1. Introduction

Heart rate (HR) and respiratory rate (RR) are important vital signs used to assess the patient’s state; normal values for these two parameters vary according to age. In adults, under resting conditions, HR ranges from 50–80 beats per minute (bpm) and RR from 12–18 breaths per minute (breaths/min). Both of these frequencies are higher in neonates: i.e., HR ranges from 120–160 bpm and RR ranges from 30–50 breaths/min [1]. Currently, assessment of HR is generally performed either by (1) measuring the electrical activity of the heart (electrocardiography; ECG); or by (2) detecting volumetric changes in blood (plethysmography). In turn, monitoring of RR is performed by (1) assessing chest or chest-abdomen movement with respiratory belt transducers [2,3]; (2) measurement of electrical impedance of the thorax; or (3) computing the direct influence of breathing on ECG morphology (ECG-derived respiratory signal) or photoplethysmography (PPG) morphology [2]. Although these monitoring techniques are reliable and not costly, they require attachment of adhesive sensors on the patient, often leading to discomfort, stress, and even pain or soreness [4]. In addition, removal of the adhesive electrodes can result in epidermal stripping, especially in premature neonates and elderly patients whose skin is fragile and easily damaged [5]. Moreover, there are more precise (albeit more invasive techniques) to measure RR, such as capnography (assessment of partial pressure of carbon dioxide in exhaled air) and spirometry (assessment of variation in air flow) [2].

There have been increasing demands for unobtrusive and contactless, as well as reliable and feasible, monitoring techniques to improve patients’ quality of life and optimize the use of medical resources [6]. Examples of new monitoring solutions that have been tested and proposed include: Doppler radar [6], capacitive electrocardiography (cECG) [7], magnetic induction [8], and imaging sensors, e.g., visible/near-infrared [2,9,10,11], mid-wave infrared (MWIR) [3,12] and long-wave infrared (LWIR) imaging sensors [3,13,14,15,16].

Infrared thermography (IRT), also known as thermal imaging, has emerged as a promising diagnostic and monitoring technique in a broad range of medical fields. For example, the use and relevance of thermal imaging has been examined for: assessment of HR [12,13] and RR [3,4,16], detection of diabetic foot ulcers [17], objective pain assessment [18], monitoring of thermoregulation in neonates [19,20], and observation of circulation and perfusion dynamics [14]. In addition, fever screening of incoming passengers at airports is a recent medical application of thermal imaging; in 2017, Sun et al. [21] introduced a new approach (which combined IRT and a CMOS camera) to screen patients with infection. Thus, in short, IRT is a remote, non-contact and passive monitoring approach, which detects the radiation naturally emitted from an object (e.g., human skin). However, the most important advantage of thermal imaging over other imaging modalities is that it does not require a radiation source [14,22].

The present study presents a new approach for contactless and passive monitoring of HR and RR using thermal imaging. This work is an extension of our earlier study which focused on the estimation of RR alone [4]. In the present study, the approach used to detect RR is based on the fact that the temperature around the nostrils fluctuates during the respiratory cycle (inspiration/expiration). The algorithm used to estimate HR is based on the cyclical ejection of blood from the heart to the head (through carotid arteries and thoracic aorta), which leads to periodic movements of the head. This hypothesis was postulated by Löser [23] as early as 1989, who called it “ballistography of the head”. Thus, the algorithm presented here detects subtle head oscillations that accompany the cardiac cycle. For a proof-of-concept, this proposed approach to assess both HR and RR by means of thermal videos was validated in a pilot study that included 20 healthy human volunteers. In this paper, Section 2 describes the developed methodology, Section 3 introduces the experimental protocol and setup, the results are presented in Section 4 and discussed in Section 5, and Section 6 presents the conclusions and some future perspectives.

## 2. Methodology

### 2.1. Respiratory Rate

**Figure 1:**
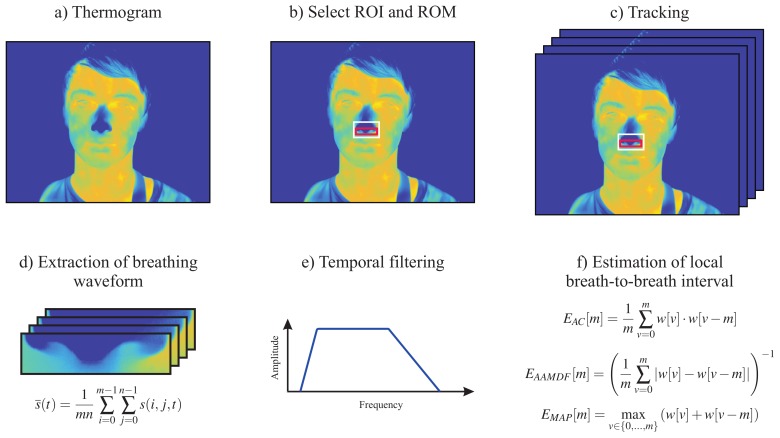
Illustration of the five major steps used to estimate respiratory rate from thermal videos of healthy adults. (**a**) Original thermogram; (**b**) Detection of the region of interest (ROI) and region of measurement (ROM); (**c**) Tracking of both ROI and ROM; (**d**) Extraction of the respiratory waveform from the ROM; (**e**) Temporal filtering using a second-order Butterworth band-pass filter; (**f**) Estimation of local breath-to-breath interval using the three estimators proposed by Brüser et al. [24].

To extract RR, we used the approach previously described by our group [4]. This is based on the fact that temperature around the nostrils decreases during inspiration (inhalation of cold air from the environment) and increases during expiration (exhalation of warm air from the lungs). Figure 1 illustrates the five main steps used to estimate RR from thermal video footage. The first step of the algorithm consisted of automatically identifying the nose, i.e., the region of interest (ROI), in the first frame of the thermal video (Figure 1b). Then, a rough tracking of the ROI was performed to compensate for the motion of the subject (Figure 1c). To improve the signal-to-noise ratio (SNR), a second ROI, called the region of measurement (ROM), was defined. The respiratory waveform corresponds to the mean temperature value s(t) of the ROM for each single frame as given by

(1) $$\bar{s}\left(t\right)=\frac{1}{mn}\sum_{i=0}^{m-1}\sum_{j=0}^{n-1}s\left(i,j,t\right).$$

Here, s(i,j,t) is the temperature at pixel (i,j) and time point *t*. The parameters *m* and *n* describe the width and length of the ROI. The respiratory waveform was further preprocessed by applying a second-order Butterworth band-pass filter with a lower and upper 3 dB cutoff frequency of 0.1 Hz and 0.85 Hz, respectively.

To estimate the instantaneous respiratory frequencies, the algorithm proposed by Brüser et al. [24] was used. This allows the accurate estimation of the local breath-to-breath and beat-to-beat intervals in physiological time signals. In this approach, an adaptive short analysis window $w_{i}\left[v\right]$ was slid across the signal s[n]. For each window position *i*, the local breath-to-breath interval $T_{i}$ was estimated using the information of three estimators: (1) *adaptive window autocorrelation* (AC); (2) *adaptive window average magnitude difference function* (AMDF); and (3) *maximum amplitude pairs* (MAP).

1. *Adaptive window autocorrelation* - $E_{AC}\left[m\right]$
  The adaptive window correlation was computed for all interval lengths (discrete lags) *m* as given by
  (2) $$E_{AC}\left[m\right]=\frac{1}{m}\sum_{v=0}^{m}w\left[v\right]\;\cdot \;w\left[v-m\right].$$
  In summary, $E_{AC}$ determines the correlation between *m* samples to the right w[v] and to the left w[v−m] of the analysis window center w[0].
2. *Adaptive window average magnitude difference function* - $E_{AMDF}\left[m\right]$
  The $E_{AMDF}$ estimator, in turn, finds the absolute difference between samples according to
  (3) $$E_{AMDF}\left[m\right]={\left(\frac{1}{m}\sum_{v=0}^{m}\left|w\left[v\right]-w\left[v-m\right]\right|\right)}^{-1}.$$
3. *Maximum amplitude pairs* - $E_{MAP}\left[m\right]$
  The third and last estimator can be interpreted as an indirect peak detector, as it only concerns the signal amplitude. $E_{MAP}\left[m\right]$ is computed as follows
  (4) $$E_{MAP}\left[m\right]=\max_{v\in \{0,...,m\}}\left(w[v]+w[v-m]\right).$$
  It achieves its maximum when two peaks, separated by *m* samples, are included in the analysis window.

To combine the estimators (which were independently computed) a Bayesian fusion method was implemented. Hence, given the three estimators $P(m|E_{AC},E_{AMDF},E_{MAP})$, the conditional probability of *m* being the correct breath-to-breath interval can be computed as follows:

(5) $$P\left(m|E_{AC},E_{AAMDF},E_{MAP}\right)\propto P\left(m|E_{AC}\right)\;\cdot \;P\left(m|E_{AAMDF}\right)\;\cdot \;P\left(m|E_{MAP}\right).$$

Note that the estimators may be considered as probability density functions [$P(m|E_{AC})$, $P(m|E_{AAMDF})$ and $P(m|E_{MAP})$] as reported in [25].

### 2.2. Heart Rate

**Figure 2:**
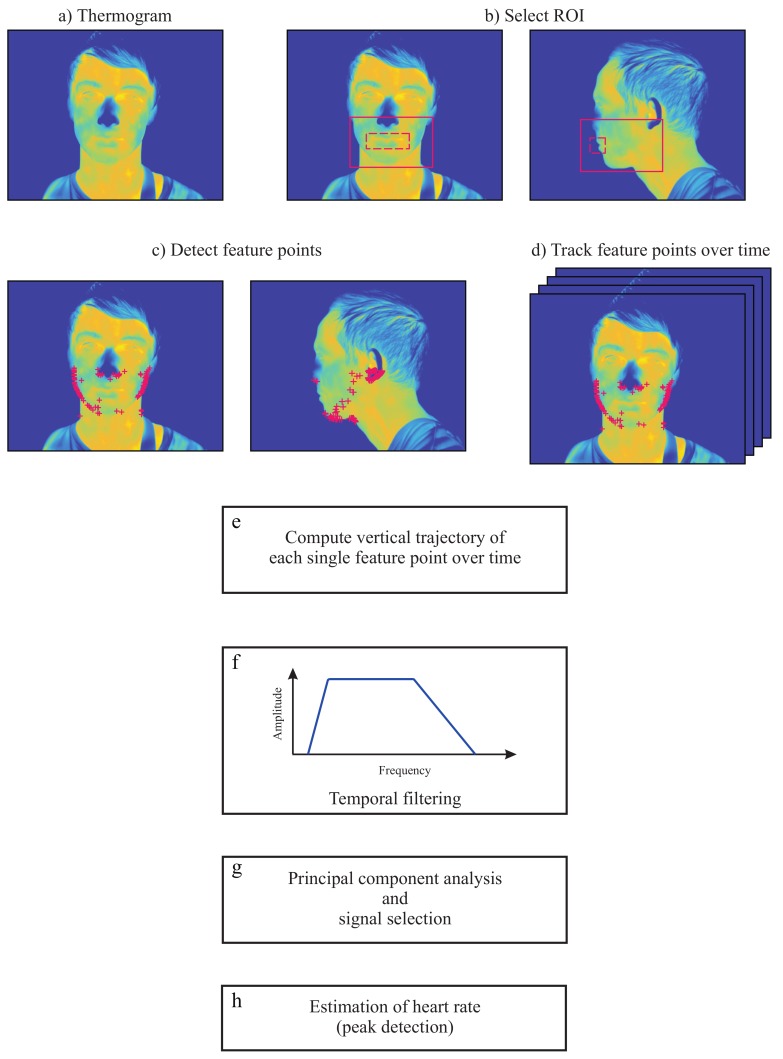
Illustration of the major steps used to estimate heart rate from thermal videos of healthy adults. (**a**) Segmentation; (**b**) Selection of region of interest (ROI) for frontal views and side views; (**c**) Detection of feature points (frontal views and side views); (**d**) Tracking of feature points; (**e**) Computation of vertical trajectories; (**f**) Temporal filtering of vertical trajectories; (**g**) Principal component analysis (PCA) and selection of the principal component; (**h**) Selection of the component with the clearest main frequency.

The approach used to estimate HR from thermal videos is based on the fact that subtle mechanical head movements accompany the cardiac cycle [26]. In fact, the feasibility of head ballistocardiography has been established for almost 30 years [23], albeit the measurements were not performed with cameras at that time. The main steps required to estimate this vital parameter with a LWIR camera are shown in Figure 2 and are described below. The algorithm was programed in MATLAB (MATLAB 2014a, The MathWorks Inc., Natick, MA, USA). Furthermore, the algorithm was tested on a 64-bit Windows 7 computer with a quad-core Intel^®^ Core™i5-3450 3.10 GHz processor, 16 GB RAM and a solid-state drive. Data were analyzed offline.

#### 2.2.1. Image Preprocessing

The first step of the algorithm consisted of preprocessing the thermograms. For that, the head of the subject was segmented from the background using a multilevel Otsu’s algorithm [27]. In short, it uses discriminant analysis to calculate optimal threshold values [27,28]. Background segmentation is relatively easy to perform in thermograms because the ambient temperature is generally lower than skin temperature. Afterwards, the contrast of the images was enhanced by linearly stretching the original gray levels to a new range. Both background subtraction and contrast enhancement were performed for each thermogram of the thermal video.

#### 2.2.2. Region Selection

Image preprocessing was followed by manual selection of the ROI in the first frame of the thermal video. The ROI should enclose only the lower portion of the head (lower part of the head beginning below the eyes) as shown in Figure 2b (red solid line). The next step was the selection of feature points within this region. However, to avoid potential artifacts, the region enclosing the mouth was neglected (dashed red line in Figure 2b). Note that no feature points were selected inside this area.

#### 2.2.3. Selection and Tracking of Feature Points

In the present study, the approach of Shi and Tomasi [29] (called the ’Shi-Tomasi corner detector’) was adopted to find optimal feature points within the ROI. This uses a scoring function *R* to ‘rate cornerness’:

(6) $$R=\min(\lambda_{1},\lambda_{2}).$$

Thus, a feature is solely considered as a corner if both eigenvalues ($\lambda_{1}$ and $\lambda_{2}$) and, consequently *R*, are greater than a threshold α: $\min(\lambda_{1},\lambda_{2})>\alpha$.

The selection of feature points was only performed in the first frame of the video sequence. The candidate features were ranked according to their strength/score [$\min(\lambda_{1},\lambda_{2})$], and only the *N* strongest features were chosen for tracking (Figure 2c). The maximum number of feature points *N* to be tracked is generally predefined by the user. Empirical evidences demonstrated that in thermal images, no more than 100 feature points are necessary.

The positions of the *N* feature points were given as input to the template-based point tracker: the *Kanade-Lucas-Tomasi tracker*. This algorithm allows to track the trajectory of all feature points (in the *x* and *y* direction) between the first and last frame of the video (Figure 2d). As the ballistocardiographic movement of the head occurs mostly in the vertical direction, only the vertical component of the position time series was used in the following steps (Figure 2e). In the present study, we assumed that some feature points may present erratic trajectories. Therefore, to preserve the most stable feature points, we omitted those feature points with trajectories between consecutive frames that surpassed a predefined percentile.

#### 2.2.4. Temporal Filtering

The fifth step of this approach consisted of temporal filtering of the vertical component trajectories. However, not all vertical trajectories are related to the beating heart. For example, low frequencies due to respiratory movements, and changes in posture, have higher amplitudes and therefore dominate the trajectories of the feature points. In a resting condition, the normal HR of an adult ranges from 45–120 bpm. Therefore, the trajectories were preprocessed by applying a Butterworth band-pass filter with a lower and upper 3 dB cutoff frequency of 0.65 Hz and 5 Hz, respectively (Figure 2f). The upper cutoff frequency was set to include the HR harmonics, since they may provide more information for correct peak detection.

#### 2.2.5. Principal Component Analysis Decomposition

The vertical movement of the head due to cyclical ejection of blood is the underlying signal of interest. However, other physiological sources which were not filtered in the previous step may also influence the trajectories of the feature points. These include, for example, vestibular activity, involuntarily muscle movements, and changes in facial expression. Within this context, it is mandatory to decompose the other motions into subsignals to isolate the heart-related component. In this study, principal component analysis (PCA) was applied to find the set of main dimensions along which the position of the head varies.

The basic idea of PCA is to diminish the dimension of a dataset, composed of interrelated variables, by maintaining the existing variation. This is achieved by transforming the initial dataset to a new set of uncorrelated variables, designated as principal components. The principal components are ordered so that the first few include most of the variation contained in the original dataset [30].

During video acquisition, head movements can occur for various reasons, such as changes in posture, swallowing, etc. Such movements might affect the position vectors by adding variance and, consequently, the PCA decomposition. To overcome this issue, it was predefined that 15% of the feature point trajectories with the largest L2-norms are not considered in the PCA.

#### 2.2.6. Principal Component Selection

Finally, the last step of this algorithm consisted of selecting the correct component/eigenvector in order to extract the pulse signal. The eigenvectors are generally ordered according to their variance. As a result, the first principal component contains the greatest percentage of variation in the data, and the *i*th principal component contains the *i*th greatest amount of variation. Empirical evidences demonstrated that, in this specific case, only the first six components are necessary for further analysis. However, the signal with the greatest variance does not necessarily contain the ballistocardiographic movements of the head. In this study, the principal component was selected based on the signal periodicity. For this, the approach presented by Li et al. [10] and Balakrishnan et al. [26] was used. The periodicity of the signal was quantified by using the peak-to-total ratio approach. This corresponds to the ratio of the frequencies within a range of 0.05 Hz around the dominant frequency and a range of 0.05 Hz around its first harmonic to the total spectral density. The amplitude spectra of the principal components were computed using the Fast Fourier Transform. Directly after selecting the component with the highest periodicity, the pulse signal was extracted. For this, the frequency spectrum was analyzed, and the clearest main frequency was chosen.

In the approach of Balakrishnan et al. [26], PCA was applied to the whole signal length of the feature point trajectories. In contrast, in the present study, a moving analysis window with a length of 256 samples (frame rate of 50 frames per second; fps) was applied. For each window position, the trajectories were filtered using a band-pass filter (Section 2.2.4), PCA was applied (Section 2.2.5) and the most periodic component selected (Section 2.2.5). Lastly, the main frequency was chosen (Section 2.2.5).

## 3. Experimental Protocol and Setup

**Figure 3:**
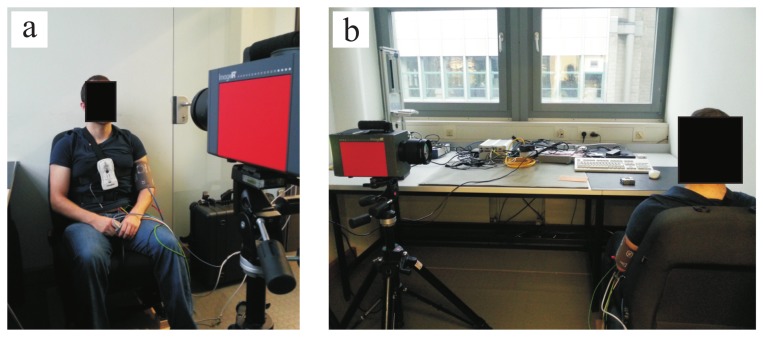
Experimental setup. (**a**) Frontal view acquisitions; (**b**) Side view acquisitions.

A total of 20 healthy volunteers (7 females, 13 males) with an average age of 27.85 years ± 6.90 years agreed to participate in this pilot study. Table 1 presents information on these volunteers (including ID number, gender and age). For the measurements, the thermal camera was fixed on a tripod placed at a distance of ± 1.5 m from the subjects. Figure 3 shows the measurement scenario. The study protocol consisted of two parts: phase A and phase B. In phase A, the volunteers were asked to sit as still as possible while looking at the camera (for frontal views; Figure 3a). During phase B, the chairs were rotated 90${}^{\circ }$ (for side views; Figure 3b). In both cases, the subjects were asked to avoid involuntary head movements.

Thermal video fragments of 3-min duration each were acquired using a high-resolution MWIR camera, ImageIR^®^ 9300 (InfraTec GmbH, Dresden, Germany). This includes a cooled InSb focal plane array photon detector with a spatial resolution of 1024 × 768 pixels. The camera detects infrared wavelengths in the spectral range of 2.0–5.5 μm and presents a thermal sensitivity better than 0.025 K at 30 ${}^{\circ }C$. In the present study, infrared thermograms were acquired with a frame rate of 50 fps.

Thoracic effort (piezoplethysmography) and PPG signal were measured simultaneously as ground truth (GT) or gold standard. The thoracic effort was assessed using the data recording system SOMNOlab 2 (Weinmann GmbH, Hamburg, Germany) with a sampling rate of 32 Hz, whereas the PPG signal was assessed using the patient monitor IntelliVue MP70 (Koninklijke Philips N.V., Amsterdam, the Netherlands) with a sampling rate of 125 Hz. During the experiments, room temperature was simultaneously measured using the data logger HOBO^®^ U12-012 (Onset Computer Corp., Bourne, MA, USA); this was (on average) 22.30 ${}^{\circ }C$.

## 4. Results

### 4.1. Respiratory Rate

Table 2 shows the performance of the algorithm developed for estimation of RR. For this measurement, data of only 16 subjects could be analyzed, i.e., because the camera was not set at the correct angle. Thus, data from subjects 2, 13, 15 and 19 were excluded as the nostrils were not clearly visible (signals from the ROIs of these four subjects contained only noise). Comparison between the two monitoring modalities (IRT and piezoplethysmography) showed a root-mean-square error (RMSE) of 0.71 ± 0.30 breaths/min. Furthermore, the mean RR error ($\bar{\varepsilon }$) was 0.03 ± 0.01 and the spread of the error, calculated using the 90th percentile of the errors ($\varepsilon_{90}$), reached 0.08 ± 0.03. On average, the RR of the subjects stayed around 15.36 ± 3.95 breaths/min. For evaluation of the algorithm, only data from phase A were used.

**Figure 4:**
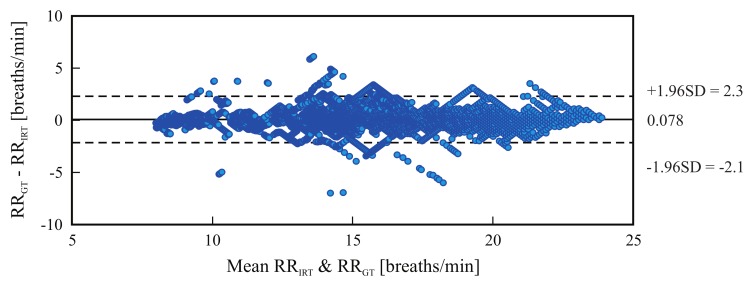
Bland-Altman plot comparing both methods, IRT-based assessment of respiratory rate (RR${}_{\mathrm{IRT}}$) and piezoplethysmography-based assessment (RR${}_{\mathrm{GT}}$); the plot comprises the data of all tested subjects. The graph shows a bias of 0.078 breaths/min (solid line) and the 95% limits of agreement range from −2.1 breaths/min to 2.3 breaths/min (dashed lines).

Figure 4 is a Bland-Altman plot comparing the two measurement techniques (IRT and ground truth) for all tested candidates. Based on the results, the estimated mean difference was 0.078 breaths/min and the limits of agreement ranged from −2.1 breaths/min to 2.3 breaths/min.

### 4.2. Heart Rate

#### 4.2.1. Frontal View

Table 3 presents the results for heart rate estimation using a frontal view acquisition. Note that the data from subjects 8 and 10 were excluded from the analysis because the ground truth consisted mainly of artifacts (thus, Figure 3 shows results for 18 subjects). The RMSE averaged 3.53 ± 1.53 bpm. Moreover, the mean HR error ($\bar{\varepsilon }$) was 0.04 ± 0.02 and the spread of the error, calculated using the 90th percentile of the errors ($\varepsilon_{90}$), reached 0.08 ± 0.03. The HR of the subjects stayed (on average) around 68.57 ± 10.52 bpm.

**Figure 5:**
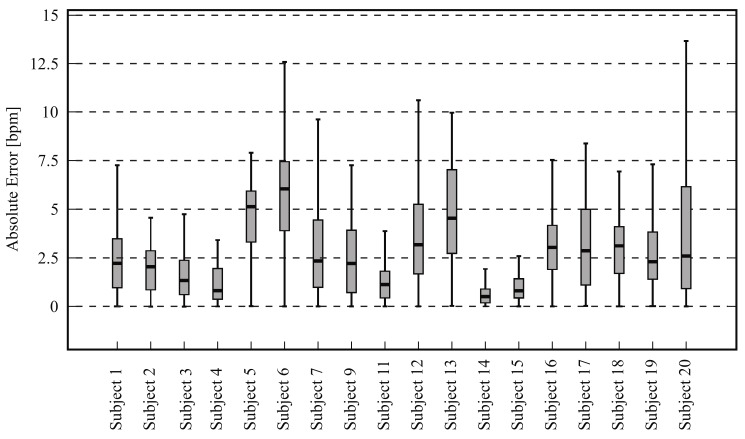
Absolute heart rate error distribution between thermal imaging and ground truth for 18 subjects; outliers were excluded.

**Figure 6:**
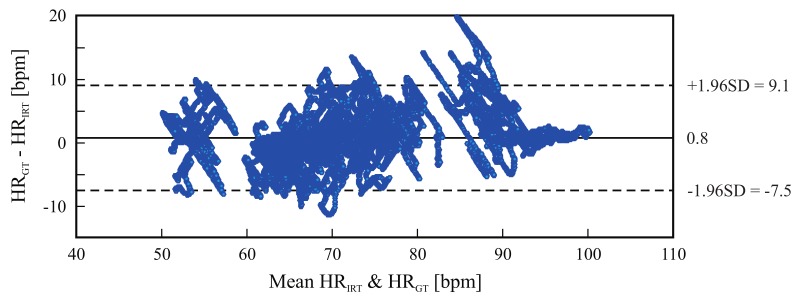
Bland-Altman plot comparing thermal imaging (HR${}_{\mathrm{IRT}}$) and ground truth (HR${}_{\mathrm{GT}}$); the plot comprises the data of 18 subjects. The graph shows a bias of 0.8 bpm (solid line) and the 95% limits of agreement range from −7.5 bpm to 9.1 bpm (dashed lines).

**Figure 7:**
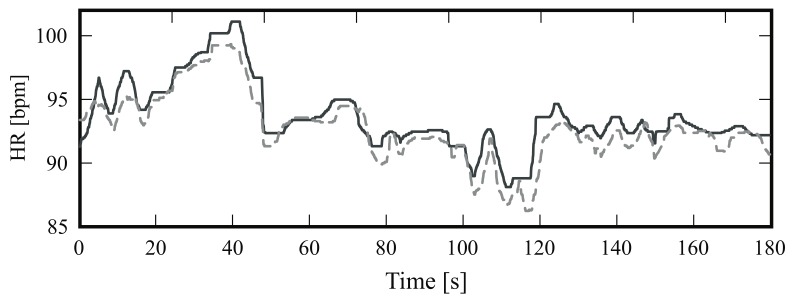
Representative example of the heart rate (HR) of subject number 15; the solid line indicates the HR obtained with photoplethysmography (PPG) and the dashed line HR estimated with infrared thermography.

Figure 5 is a boxplot diagram showing the absolute HR error distribution for each participant; outliers were excluded from this analysis. Figure 6 is a Bland-Altman plot comparing the two measurement techniques (IRT and ground truth) for all subjects. Here, the estimated mean difference was 0.8 bpm and the limits of agreement ranged from −7.5 bpm to 9.1 bpm. Finally, Figure 7 presents an example of HR estimated with thermal imaging (dashed line) as well as the HR corresponding to the ground truth (solid line); these representative signals are from subject number 15.

#### 4.2.2. Side View

Table 4 presents the results for HR estimation using side view acquisitions. Note that the data from subjects 3, 5, 10 and 20 could not be evaluated. Due to technical difficulties, the ground truths from subjects 3, 5 and 20 were not recorded, and the PPG signal from subject 10 contained many artifacts; therefore, only 16 datasets were available. Comparison between the two monitoring modalities (PPG and thermal imaging) showed a RMSE of 3.43 ± 1.61 bpm. In addition, the mean HR error ($\bar{\varepsilon }$) was 0.04 ± 0.01 and the spread of the error, calculated using the 90th percentile of the errors ($\varepsilon_{90}$), stayed around 0.08 ± 0.03. On average, the HR of the subjects was 67.27 ± 10.06 bpm.

**Figure 8:**
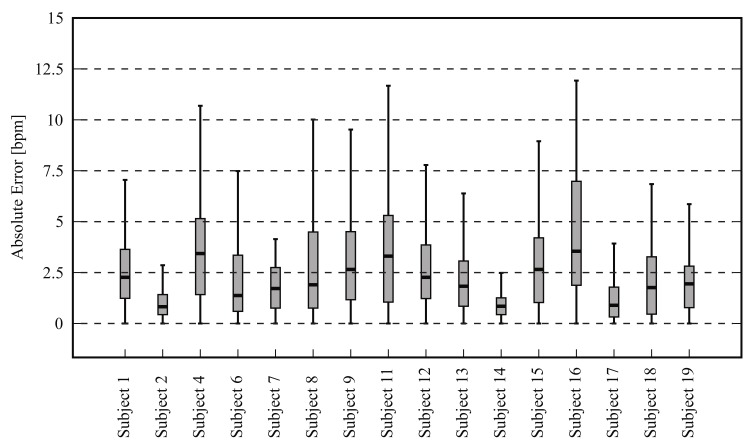
Boxplot showing the absolute heart rate error distribution between thermal imaging and the gold standard for 16 subjects (side views); outliers were excluded.

**Figure 9:**
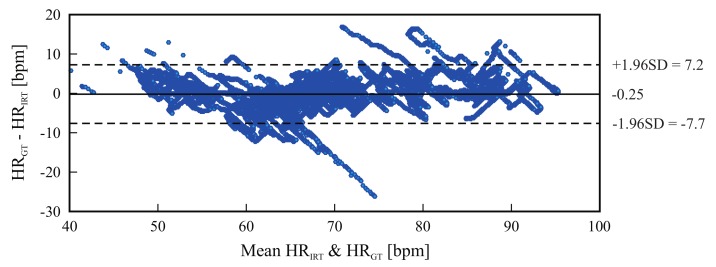
Bland-Altman plot comparing thermal imaging (HR${}_{\mathrm{IRT}}$) and photoplethysmography (HR${}_{\mathrm{GT}}$); the plot comprises the data of 16 subjects. The graph shows a bias of −0.25 bpm (solid line) and the 95% limits of agreement range from −7.7 to 7.2 (dashed lines).

**Figure 10:**
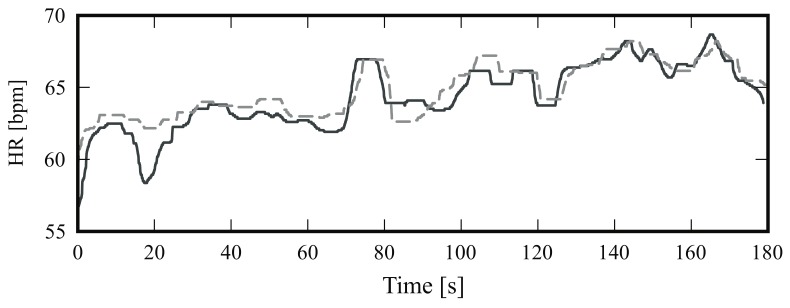
Estimated heart rates. The solid line indicates the heart rate obtained with photoplethysmography, and the dashed line indicates the HR estimated with infrared thermography; these signals are derived from subject number 2.

Figure 8 shows the absolute HR error distribution for each volunteer; again, the outliers were not included. Figure 9 is a Bland-Altman plot comparing the two measurement techniques (IRT and ground truth) for all 16 candidates. Here, the bias was −0.25 bpm and the limits of agreement ranged from −7.7 bpm to 7.2 bpm. Lastly, Figure 10 shows the HR estimated with thermal imaging (dashed line) and with PPG (solid line); the signals of this representative example are from subject number 2.

## 5. Discussion

Heart rate and respiratory rate are essential parameters for patient assessment. However, current measurement techniques require the attachment of sensors to the patients body, leading to discomfort, stress and even pain. We have presented a new approach for noninvasive and totally passive monitoring of both HR and RR rate using thermal imaging. The RR algorithm is based on temperature fluctuations around the nostrils during the respiratory cycle (inspiration/expiration), whereas the HR algorithm is based on the cyclical ejection of blood flow from the heart to the head, through the carotid arteries and thoracic aorta, that generally lead to periodic vertical movements of the head. To test the performance of our approach, a pilot study was conducted with 20 healthy volunteers. This was carried out under ideal conditions, i.e., the subjects were instructed to sit as still as possible during video acquisition to reduce the amount of motion artifacts. Each subject underwent two measurements: in phase A frontal view videos were recorded, and in phase B side views were recorded (i.e., a total of 40 recordings for the 20 subjects). Since the RR approach has already been validated in a similar way in [4], this discussion focuses mainly on the performance of the HR algorithm.

For assessment of RR, only the videos acquired in phase A were used. However, not all measurements could be considered in this validation. In the thermal videos of subjects 2, 13, 15 and 19 the nostrils were not visible because the camera orientation was above the subject’s eye level (high angle acquisition). For the remaining 16 subjects, Table 2 shows excellent agreement between IRT and piezoplethysmography, with a RMSE of 0.71 ± 0.30 breath/min and a mean relative error of 0.03 ± 0.01. These results are similar to those reported previously [4,31].

For HR monitoring, a comparison was made between two different image acquisitions (frontal and side view videos). In side views, the contour of the chin is more pronounced due to the contrast between the foreground and background. In frontal views, the contrast between chin and neck is less prominent since they both present a similar temperature. Therefore, we analyzed both image acquisitions in order to examine whether side views might offer more accurate results.

Unfortunately, in this validation, not all thermal videos could be considered. For frontal acquisitions, data from subjects 8 and 10 had to be excluded as the ground truth contained many artifacts. For side acquisitions, only 16 datasets were available, i.e., due to technical difficulties, the ground truth from subjects 3, 5 and 20 were not recorded. In addition, the PPG signal from subject 10 contained many artifacts.

For the recordings of both views, good results were obtained. Table 3 and Table 4 show excellent agreement between thermal imaging and the reference. The average RMSE for frontal acquisitions was 3.53 ± 1.53 bpm; the mean relative HR error was 0.04 ± 0.02 and the spread of error reached 0.08 ± 0.03. Similar results were obtained for the side view recordings; the RMSE stayed around 3.43 ± 1.61 bpm. The same applies to the mean HR relative error and the spread of the error; the former averaged 0.04 ± 0.01 and the latter was 0.08 ± 0.03. In both video acquisitions, the minimum error was found for subject 14. We believe that the strength of the ballistocardiographic movement differs between subjects, i.e., they can be affected by the subject’s anatomy, body mass index, posture during the measurement, age and (perhaps) even to gender.

The boxplots in Figure 5 and Figure 8 illustrate in detail the absolute error distribution for each volunteer; obviously, the performance of the algorithm differed between subjects. For frontal views, the best agreement was obtained for subjects 14 and 15. Figure 7 presents the HR signals from subject 15; here the excellent agreement between the two modalities is corroborated. The highest errors were obtained for subjects 6 and 20. For side views, the best results were achieved for subjects 2 and 15 (Figure 8 and Figure 10), whereas poor agreement was obtained for subjects 11 and 16. However, the present results may have been influenced by a variety of factors, including the contrast and quality of the thermal videos, the anatomy of the subjects, and movement artifacts. Although the volunteers were requested to sit as still as possible, some involuntary movements could not always be avoided (e.g., swallowing, smiling, etc.).

Figure 6 and Figure 9 are Bland-Altman plots for both measurements, with the data of all tested volunteers. The plots show good agreement between IRT and the reference. The accuracy was very good: the bias stayed around 0.8 bpm and −0.25 bpm for the frontal views and side views, respectively. However, IRT imaging may be more accurate when using side view acquisitions. Additionally, the 95% limits of agreement confirm the high precision of this measurement technique. For frontal views, more errors were observed with increased HR (80-90 bpm). In summary, the Bland-Altman diagrams, together with Table 3 and Table 4, illustrate the capability of this approach to accurately estimate a broad range of HRs, ranging from approximately 40–100 bpm.

As mentioned in Section 1, the algorithm for HR assessment was based on the work of Balakrishnan et al. [26]; their approach was validated using recordings from their 18 healthy subjects (7 females and 11 males), aged 23–32 years. Their videos were acquired with a regular camera (Panasonic Lumix GF2) in a room with sufficient ambient light. To compute the mean pulse rate, the authors used the frequency of maximal power for the selected PCA component. However, in contrast to our approach, PCA was applied to the whole length of the feature point trajectories. The reference pulse rate, in turn, stood for the main frequency of the ECG spectrum. By comparing the mean HR, a mean relative error of 1.5% was achieved by Balakrishnan and et al. [26]. Similar results were obtained in the present study: i.e., by comparing only the mean HR, the mean relative errors averaged 2.1% and 2.3% for the frontal and side views, respectively.

Other groups have also reported approaches for the assessment of HR using IRT. For example, in 2007, Garbey et al. [12] presented a novel method to measure cardiac pulse. This was based on information contained in the thermal signal emitted from major superficial vessels, as their temperature is modulated by pulsating blood flow. To estimate the pulse, a line-based region along the vessel was extracted. The authors focused on vessels such as external carotid, superficial temporal and artero-venous complex. For their measurements, the authors used a MWIR camera with an indium antimonide high-efficiency photon detector. This yielded a temperature resolution smaller than 25 mK and was capable of capturing 30 fps in full spatial resolution (640 × 480 pixels). To evaluate the algorithm, 34 thermal clips from 34 healthy volunteers were recorded. The results obtained with thermal imaging (TI) were compared with a GT using a measure denominated complement of the absolute normalized difference (CAND):

(7) $$CAND=1-\frac{\left|GT-TI\right|}{GT}.$$

The evaluation showed a mean CAND of 88.53%. By considering only those subjects with a clear vessel thermal imprint (21 of 34 subjects), the authors achieved an increase of the CAND to 90.33% was observed. With our approach, using the same comparison method (CAND), better results were obtained, i.e., 97,87% for frontal acquisitions and 97.66% for side acquisitions. This may indicate that motions due to pulsating blood are easier to detect than the thermal signal emitted from major superficial vessels.

In 2006, Chekmenev et al. [13] proposed a similar approach to that of Garbey et al. [12]. For the video recording, a LWIR camera with a thermal sensitivity of 25 mK and a spatial resolution of 320 × 256 pixels was used. In addition, the thermal recordings were acquired at a rate of 30 fps and had a duration of approximately 20–40 s. Their approach was tested on videos from five healthy subjects (4 males, 1 female; aged 24–35 years). The authors reported 100% accuracy of the method when the ROI enclosed the vessels of interest, e.g., the carotid artery area.

It is important to emphasize that several factors can influence the performance of the algorithm used to assess the cardiac pulse, i.e., (1) major movement artifacts; (2) the number; and (3) the position of feature points, as well as (4) the length of the moving window. In their study, Balakrishnan et al. [26] used 1500 feature points. In our approach, 100 points were sufficient to obtain a considerable trade-off between computation time and algorithm performance. In infrared images, the ROI must only include the lower part of the head. By selecting the whole head as ROI, the greater part of the feature points were defined in the region of the forehead, due to the high contrast of the hair. On the other hand, by choosing the lower part of the head, feature points were mainly located on the maxillary contour. Finally, the length of the moving window plays a major role on algorithm outcome: a window size of 256 samples (based on a sample rate of 50 fps) showed the best trade-off between both spectral and temporal resolution.

As mentioned in Section 2, our ROIs were selected manually. Automatic detection can be achieved using the Viola-Jones algorithm, a machine learning approach for visual object detection using the Haar feature-based cascade classifier. The OpenCV and MATLAB Viola-Jones algorithms are already trained to detect faces in visual images; however, for thermal images, the classifier must be trained again.

Moreover, the room/air temperature (as well as other direct environmental conditions) can affect the accuracy of thermography. In contrast with other studies (which focused primarily on body temperature and its distribution) the present study does not rely on the capability of the camera to accurately measure an absolute temperature. The main factors affecting the performance of our approach are the resolution of the focal plane array and the thermal sensitivity of the camera.

In future studies, the ability of the algorithm to measure chest surface motion due to heart beat should be investigated. Shafiq et al. [32] reported that, in adults, weaker vibrations (precordial motion) caused by heartbeat and by respiration result in a chest surface motion ranging from 0.2–0.5 mm and from 4–12 mm, respectively.

## 6. Conclusions

An approach has been presented for unobtrusive monitoring of two vital parameters, HR and RR, using IRT. This is based on temperature fluctuations around the nose and on the cyclical ejection of blood flow from the heart, which leads to periodic movements of the head in the vertical direction.

To test the feasibility of this approach, a pilot study was conducted with 20 healthy adults in which two acquisition views were tested, i.e., frontal and side views. In general, the results show considerable agreement between IRT and the gold/reference standards. For HR, the RMSE stayed around 3 bpm. For RR, the RMSE between thermal imaging and piezoplethysmography averaged 0.7 breaths/min.

However, despite these promising results, some improvements are still required. A future aim should be to integrate a motion analysis algorithm capable of automatically detecting motion artifacts in the thermal video. Furthermore, the capability of this technique to measure chest surface motion due to heartbeats should be explored.

Thermal imaging might be a suitable alternative for monitoring cardiorespiratory signals. In fact, this technique is ideal for long-term monitoring, screening purposes, automotive applications, and integration in domestic sites.

## Figures and Tables

**Table 1:** Subject data: ID number, gender (F, female; M, male) and age.

Subject ID	Gender	Age (Years)
Subject 1	M	36
Subject 2	M	30
Subject 3	M	25
Subject 4	F	33
Subject 5	F	25
Subject 6	M	23
Subject 7	M	27
Subject 8	M	29
Subject 9	M	25
Subject 10	F	36
Subject 11	M	25
Subject 12	M	27
Subject 13	F	31
Subject 14	M	50
Subject 15	M	18
Subject 16	M	19
Subject 17	F	23
Subject 18	F	23
Subject 19	F	26
Subject 20	M	26
**Mean ± SD**		**27.85 ± 6.90**

**Table 2:** Results for respiratory rate estimation (*n* = 16) using frontal view acquisitions.

Subject	Mean HR		RMSE (Breaths/Min)	$\bar{\varepsilon }$	$\varepsilon_{90}$
	GT (Breaths/Min)	IRT (Breaths/Min)			
Subject 1	13.94	13.51	1.28	0.07	0.13
Subject 3	15.46	15.34	0.46	0.02	0.05
Subject 4	15.52	15.15	0.96	0.05	0.09
Subject 5	13.10	13.16	0.59	0.03	0.08
Subject 6	20.62	20.73	0.57	0.02	0.06
Subject 7	9.04	9.10	0.24	0.02	0.05
Subject 8	12.78	12.43	0.62	0.03	0.05
Subject 9	14.21	14.13	0.58	0.03	0.06
Subject 10	18.15	17.77	1.13	0.05	0.11
Subject 11	19.39	19.43	0.70	0.03	0.05
Subject 12	9.70	9.62	0.77	0.05	0.14
Subject 14	8.79	9.10	0.81	0.05	0.10
Subject 16	17.33	17.26	1.52	0.07	0.15
Subject 17	19.33	19.57	0.74	0.03	0.07
Subject 18	21.20	21.09	0.48	0.02	0.04
Subject 20	15.71	15.81	0.47	0.02	0.04
**Mean ± SD**	**15.36 ± 3.95**	**15.31 ± 3.93**	**0.71 ± 0.30**	**0.03 ± 0.01**	**0.08 ± 0.03**

GT—ground truth, $\bar{\varepsilon }$—mean relative error, $\varepsilon_{90}$—90th percentile of the relative errors.

**Table 3:** Results for heart rate estimation (*n* = 18) using frontal view acquisitions.

Subject	Mean HR		RMSE (bpm)	$\bar{\varepsilon }$	$\varepsilon_{90}$
	GT (bpm)	IRT (bpm)			
Subject 1	72.29	71.21	4.15	0.05	0.11
Subject 2	63.26	64.37	2.20	0.03	0.06
Subject 3	74.48	73.44	1.91	0.02	0.04
Subject 4	63.54	63.98	1.80	0.02	0.05
Subject 5	63.68	67.25	4.40	0.06	0.09
Subject 6	90.46	85.22	7.16	0.07	0.14
Subject 7	73.62	73.83	3.84	0.04	0.08
Subject 9	80.46	77.65	4.46	0.05	0.10
Subject 11	68.84	69.76	2.17	0.02	0.05
Subject 12	66.79	68.06	4.66	0.06	0.11
Subject 13	55.50	53.67	5.32	0.09	0.02
Subject 14	63.33	63.91	1.13	0.01	0.03
Subject 15	93.80	92.88	1.40	0.01	0.02
Subject 16	63.54	64.65	4.07	0.05	0.11
Subject 17	66.29	66.98	3.34	0.04	0.09
Subject 18	54.07	52.10	3.49	0.06	0.10
Subject 19	59.28	59.36	3.04	0.04	0.08
Subject 20	61.05	62.80	5.01	0.05	0.11
**Mean ± SD**	**68.57 ± 10.52**	**68.40 ± 9.72**	**3.53 ± 1.53**	**0.04 ± 0.02**	**0.08 ± 0.03**

GT—ground truth, $\bar{\varepsilon }$—mean relative error, $\varepsilon_{90}$—90th percentile of the relative errors.

**Table 4:** Results for heart rate estimation (*n* = 16) using side view acquisitions.

Subject	Mean HR		RMSE (bpm)	$\bar{\varepsilon }$	$\varepsilon_{90}$
	GT (bpm)	IRT (bpm)			
Subject 1	66.69	66.81	2.87	0.04	0.06
Subject 2	63.98	64.48	1.22	0.02	0.03
Subject 4	62.11	65.84	5.77	0.06	0.13
Subject 6	87.86	85.58	4.35	0.03	0.07
Subject 7	70.03	68.72	2.24	0.03	0.05
Subject 8	65.97	66.21	3.84	0.04	0.09
Subject 9	78.77	76.81	3.95	0.04	0.08
Subject 11	65.47	68.13	6.94	0.06	0.12
Subject 12	69.82	69.51	3.71	0.04	0.08
Subject 13	54.03	52.97	2.54	0.04	0.09
Subject 14	64.36	64.86	1.08	0.01	0.02
Subject 15	88.98	86.98	4.36	0.04	0.08
Subject 16	60.02	63.99	5.18	0.07	0.14
Subject 17	65.79	66.16	1.39	0.02	0.04
Subject 18	51.08	49.43	2.68	0.04	0.10
Subject 19	61.32	63.33	2.72	0.03	0.07
**Mean ± SD**	**67.27 ± 10.06**	**67.49 ± 9.32**	**3.43 ± 1.61**	**0.04 ± 0.01**	**0.08 ± 0.03**

GT—ground truth, $\bar{\varepsilon }$—mean relative error, $\varepsilon_{90}$—90th percentile of the relative errors.
